# How do we know whether treatment has failed? Paradoxical outcomes in counseling with young people

**DOI:** 10.3389/fpsyg.2024.1390579

**Published:** 2024-06-04

**Authors:** John McLeod, Erik Stänicke, Hanne Weie Oddli, Stephanie Smith, Peter Pearce, Mick Cooper

**Affiliations:** ^1^Institute for Integrative Counselling and Psychotherapy, Dublin, Ireland; ^2^Department of Psychology, University of Oslo, Oslo, Norway; ^3^Research and Policy, National Children’s Bureau, London, United Kingdom; ^4^Faculty of Applied Social and Organisational Sciences, Metanoia Institute, London, United Kingdom; ^5^School of Psychology, University of Roehampton, Roehampton, United Kingdom

**Keywords:** paradoxical outcome, assessment, self-report, qualitative interviews, humanistic counseling

## Abstract

**Background:**

In both routine practice contexts and research studies, evidence from standardized self-report symptom measures, administered pre- and post-treatment, is predominantly used to determine whether psychotherapy has been successful. Understanding the nature of unsuccessful psychotherapy requires an ability to evaluate the credibility of outcome data generated by such techniques. An important body of research has identified discrepancies between outcomes assessed through symptom measures and those obtained from other sources. However, not enough is known about the extent to which such *paradoxical outcomes* exist.

**Objective:**

This study analyzes the relationship between outcomes, as assessed by a standardized self-report measure, and as assessed by ratings of young people’s descriptions of change at post-counseling interviews.

**Methods:**

Participants were 50 young people (13–16 years old) who had taken part in a trial of up to 10 weeks of school-based humanistic counseling. Our primary standardized measure was the Young Person’s CORE (YP-CORE). To assess young people’s experiences of counseling change, three independent raters scrutinized transcripts of post-counseling interviews, and scored levels of helpfulness on a 1 (Not at all helpful) to 10 (Extremely helpful) scale. Inter-rater reliabilities were 0.94 (Cronbach’s Alpha) and 0.96 (McDonald’s Omega). Sensitivity analyses were conducted to explore relationships between helpfulness ratings and other outcome measures, i.e., satisfaction with counseling (ESQ) and the Goal-Based-Outcome Tool (GBO), and process measures, i.e., the Working Alliance Inventory (WAI-S) and the Barret Lennard Relationship Inventory (BLRI).

**Results:**

Multilevel analysis indicated that helpfulness ratings were not significantly associated with changes in YP-CORE scores. Analyzed categorically, 38% of those showing reliable improvement on the standardized measure were below the median for self-described helpfulness, and 47% of those not showing reliable change were at or above the median for self-described helpfulness. Sensitivity analyses demonstrated closer correlations between helpfulness ratings and other outcome measures (ESQ and GBO), and between helpfulness ratings and process measures (WAI-S and BLRI).

**Discussion:**

Our results raise questions about reliance on symptom change outcome measures for defining treatment success and failure, given their disparity with clients’ own descriptions of the helpfulness of therapy. Implications for practice and research are discussed.

## Clinical significance

The capacity to review progress in therapy represents a key area of professional competence, particularly in relation to working with clients whose treatment is not on track. Evidence around the proportion of cases that report successful or unsuccessful outcomes, also makes it possible to design services in ways that are responsive to client or service user need. The findings of this study suggest that it is neither ethically nor scientifically justifiable to base such judgments solely on evidence from standardized self-report symptom measures. It is essential, instead, that both individual clinicans and service-provider operations should adopt strategies, appropriate to their client population, to take account of multiple sources of information about outcomes. Further research is required to support innovation and guideline development in this area of practice.

## Introduction

For a significant proportion of clients and patients, psychotherapy does not result in meaningful improvement in their lives. Many decades of research and practice innovation in the field of psychotherapy have adopted a primary focus on the question of how treatment can be made more effective. Although such endeavors remain important, there is also a growing appreciation that the benefits of therapy, at both individual and societal levels, require a better understanding of the nature of unsuccessful psychotherapy (Oasi and Werbart, 2020; Krivzov et al., 2021; Gazzola and Iwakabe, 2022; Paveltchuk et al., 2022; Suárez-Delucchi et al., 2022; Knox et al., 2023). Investigation of this topic has encompassed multiple lines of inquiry, including single case studies, qualitative studies, and analysis of large data sets. Across this literature, a common theme has been the analysis of data from standardized, nomothetic client self-report symptom scales, administered prior to entering therapy, over the course of psychotherapy, and at follow-up. This has also represented a key methodological strategy in relation to the study of unsuccessful therapy. Such an approach affords a rigorous and cost-effective method for differentiating between good and poor outcomes, and has been widely utilized not only for research purposes, but also as a means of obtaining feedback about client progress that can inform routine practice.

There exists a broad consensus around the ethical requirement for any symptom measure used in psychotherapy research and practice to be supported by validity and reliability data around the use of that tool as an adequate indicator of the severity of psychological difficulties and distress. However, despite the extensive research and development work that underpins such measures, there has been a growing appreciation of their limitations in the specific context of evaluating change in psychotherapy. Specifically, research has shown discrepancies between outcome profiles generated by the use of pre- and post-therapy symptom measures, and those derived from qualitative interviews conducted with the same clients [see, for example, McElvaney and Timulak (2013), Bloch-Elkouby et al. (2019), and De Smet et al. (2019, 2020a,b, 2021a,b, 2024)]. The lack of convergence between narrative accounts of outcomes, and outcome analyses based on responses to standardized measures, was described by Stänicke and McLeod (2021) as *paradoxical outcomes*, in the sense of confronting researchers with an apparent contradiction: how can it be, that different but equally credible methods of assessing outcome, can produce (in some instances) radically different conclusions?

There are several factors that may contribute to the occurrence of paradoxical outcomes. When evaluating the effectiveness of therapy they have received, clients make reference to a much wider range of criteria than those covered by commonly-used symptom measures (Chevance et al., 2020; Bear et al., 2021; Housby et al., 2021; Krause et al., 2021; Axelsdóttir et al., 2022; Morton et al., 2022; Amin Choudhury et al., 2023; Kohne et al., 2023; Krause et al., 2024). As a consequence, in a post-therapy interview, a client may judge therapy to be successful or otherwise on the basis of factors that are not measured in an outcome scale. For example, a client may talk about how helpful it was for them that therapy enabled them to re-connect with their spirituality – a dimension rarely included, or only tangentially referred to, in symptom scales.

It is also possible that the experience of engaging in therapy has the effect of leading clients to interpret items on a symptom measure in a different way: patients may respond to the same questionnaire differently after therapy because they have “recalibrated” the range of felt suffering and/or they have “reconceptualized” their symptoms (Golembiewski et al., 1976). This phenomenon has been described as *response shift* or *lack of measurement integrity* (Howard and Daily, 1979; Fokkema et al., 2013; Bulteau et al., 2019; Sawatzky et al., 2021; Verdam et al., 2021; Bulteau et al., 2023). Some studies of response shift have found that, over the course of therapy, clients develop a more differentiated and coherent understanding of the constructs being measured in outcome scales, such as anxiety or depression. As a consequence, both their end of therapy scores, and how they evaluate outcome in the context of an interview, are likely to more accurately reflect their actual distress and recovery, whereas their pre-therapy symptom scores are likely to be less reliable. A different form of response shift can occur in individuals who have learned to cope with adverse life experience by warding off painful memories and emotions, and portraying themselves as well-adjusted and resourceful – a pattern that Shedler et al. (1993) characterized as “illusory mental health.” Clients who fall into this category are likely to significantly under-report psychological symptoms in measures completed pre-therapy, and then record higher scores as their experience in therapy enables them to be more open to acknowledging personal difficulties. A paradoxical pattern is then observed in week-by-week symptom scores – as therapy becomes more successful in allowing disavowed issues to be addressed, the client appears to get worse (Ward and McLeod, 2021).

A further aspect of the measurement process that may contribute to paradoxical outcome is related to how clients interpret instructions on symptom measures. McLeod (2021) has suggested that self-report symptom measures are designed on the assumption that the respondent is able to think straight and follow instructions. The circumstances of completing a measure as a client seeking or receiving treatment are not necessarily consistent with such assumptions. In early or pre-therapy assessment, it may be hard for a client to respond accurately to the instruction to report on how they have felt over the previous week or month, because they lack any obvious way of anchoring their estimate of what their mental state was like at that earlier point. By contrast, during therapy and at follow-up, the client can refer to how they felt at the previous or final session (McLeod, 2001). Other clients may struggle to answer questions on how they “generally” feel, because their everyday experience of distress involves oscillating between contrasting emotional states, or is highly contingent on specific triggering events. Answers to questionnaire items may be idiosyncratic or skewed in clients for whom the task of completing a measure, or participating in research, has personal or emotional meaning. These micro-processes have been reported in several studies in which clients have been interviewed around their experience of completing a symptom measure (Blount et al., 2002; Galasiński and Kozłowska, 2013; Truijens, 2017; Truijens et al., 2019a,b, 2023).

It can also be hypthesised that disparities may exist between what a client says in a post-therapy interview and analysis of data from pre- and post-therapy measures, because of the limitations of qualitative methodology. Prior to such an interview, the client may have had few, if any, opportunities to review and evaluate the outcomes of their therapy. Being asked, in an interview, to make a retrospective comparison between how one feels now, compared to a pre-therapy point in time weeks or even months earlier, represents a highly demanding cognitive task. In addition, particularly if the interviewer is known to be a therapist or believed to have allegiance to the work offered even obliquely, there may be implicit pressure to provide a socially desirable account of how beneficial therapy has been. Although strategies have been developed to support clients being interviewed to look at their therapy experience in a systematic manner that invites attention to alternative perspectives [see, for example, Elliott (2002) and Sandell (2015)], it is difficult to determine how effective these approaches have been in relation to ensuring the credibility of qualitative outcome evaluations.

A range of plausible and heuristically generative theoretical frameworks for conceptualising discrepant or paradoxical outcome evaluations are discussed by Georgaca (2021), Stänicke and McLeod (2021) and Wahlström (2021). One way of making sense of the range of perspectives that exist around this topic is to differentiate between outcome evaluation strategies based on measuring distress at multiple points in time, and approaches that retrospectively invite the client to report on their subjective perception of how they have changed. Flückiger et al. (2019) suggest that there are many methodological issues associated with repeated measurement of psychological states. By contrast, inviting clients to retrospectively rate their subjective experience of change, following completion of therapy, represents a potentially valuable strategy for distinguishing between successful and unsuccessful cases (Willutzki et al., 2013). Other explanations of paradoxical outcome make connections between this phenomenon and fundamental therapeutic processes, rather than methodological considerations arising from the use of different data collection approaches. For example, Fonagy et al.’s (2015, 2017) theory of epistemic trust suggests that, at least in some instances, a patient’s response on a questionnaire may be affected by their degree of trust in the perspective on the world being offered by their therapist. As epistemic trust grows, a patient may become more able to respond authentically and accurately to items on a measure.

An important emerging strand of research into paradoxical outcome has been studies in which clients are invited to provide their own interpretation of the change profile generated by their responses to symptom measures (Roubal et al., 2018; Ghelfi, 2021; Ogles et al., 2022; Hickenlooper, 2023). The studies have consistently found that apparent discrepancies between clients’ outcome scores, and their accounts of change provided in interviews, can be readily explained by clients in terms of what was happening for them at different points in the process of therapy. In addition, clients participating in studies where they were invited to comment on their change profile, reported that this opportunity was highly meaningful for them, as a means of reflecting on and consolidating what they had learned during therapy.

With the partial exception of analysis of large therapy data sets from a response shift perspective, the potential sources of contradictory outcome assessment outlined above have only been investigated in a limited number of studies based on single cases or small sample sizes. As a result, at the present time it is not possible to assess how pervasive the phenomenon of paradoxical outcome might be, and how much of a threat it represents in relation to confidence in the credibility of analyses of therapy success derived from data obtained through self-report symptom measures. For example, interviews where clients, categorized as poor outcome cases on a routine outcome measure, describe some marginal benefits from the therapy they have received, do not necessarily undermine the conclusion that their therapy had, on balance, been unsuccessful. Conversely, the overall meaning of a study is not necessarily undermined when clients who recorded good outcomes and then tell an interviewer that while they felt that therapy had been largely successful for them, they were nevertheless disappointed that certain issues had not been addressed (Nilsson et al., 2007).

The present study examines the pervasiveness of paradoxical outcomes by mapping their occurrence in data generated by a large-scale randomized clinical trial of psychotherapy outcome. An exploratory mixed-methods secondary analysis was carried out on an existing dataset to examine the extent to which discrepancies occurred between symptom self-report and narrative self-report estimates of the successfulness and unsuccessfulness of therapy.

## Methods

### Design

This study was a secondary analysis of data collected as part of a two-arm, individually randomized trial comparing short-term (average 8 session) humanistic counseling plus pastoral care as usual versus pastoral care as usual for young people (aged 13–16 years old) with emotional symptoms ETHOS trial: Stafford et al., 2018; Cooper et al., 2021. The study was conducted in 18 schools in England (typical age range: 11–18 years old). This secondary analysis was not pre-registered.

Ethical approval for the trial was obtained under procedures agreed by the University Ethics Committee of the University of Roehampton, Reference PSYC 16/227, 31st August 2016. Young people and parents/carers advised at all stages of the study [Supplemental Material: Patient and Public Involvement].

Further information on the primary study is available in published reports on the overall findings (Cooper, 2021; Cooper et al., 2021), qualitative analysis of experiences of clients receiving counseling (Raynham et al., 2023; Cooper et al., 2024), interviews with parents and carers (Longhurst et al., 2022), and single case analyses of poor outcome cases (Ralph and Cooper, 2022; Pattison and Cooper, 2024). The overall picture that emerged from these analyses was that counseling was generally viewed as valuable by clients and their families. Typically, clients described long-term improvements in their relationships and their capacity to engage in school work, alongside reductions in emotional distress. A few clients reported that their counseling had not been benefical because they had felt awkward during sessions, for instance if there were long silences. In terms of outcomes assessed by standardized measures, the addition of humanistic counseling to routine pastoral care was associated with a higher level of symptom reduction. In interviews, some clients and their carers/parents suggested that they would have preferred a more active therapy approach, and more sessions.The findings of this study have significant policy implications in relation to the provision of school-based counseling in England. The secondary analysis reported in the present paper focuses primarily on the degree of convergence between the estimation of therapy successfulness and unsuccesfulness based on data from the primary outcome measure, the Young Person’s Clinical Outcomes in Routine Evaluation scale (YP-CORE), and the picture emerging from qualitative interviews with clients.

### Participants

#### Young people

Eligible participants were aged 13–16 years old and experiencing moderate to severe levels of emotional symptoms [as indicated by a score of 5 or more on the Emotional Symptoms subscale of the self-report Strengths and Difficulties Questionnaire, SDQ-ES, range = 0–10, Goodman (2001)]. They had an estimated English reading age of at least 13 years, wanted to participate in counseling, had a school attendance record of 85% or greater (to increase likelihood of attending testing meetings), and were not currently in receipt of another therapeutic intervention. Exclusion criteria were: incapable of providing informed consent for counseling, planning to leave the school within the academic year, and deemed at risk of serious harm to self or others.

Participants for the full trial were recruited from 18 state-funded schools in the Greater London area (typical age range 11–18 years old). The research team conducted 596 assessments for the trial, yielding 330 cases. Qualitative interviews were conducted with a sample of young people from nine of the schools, selected to maximize representativeness across the full sample. In total, 53 young people assented to be interviewed (31.7% of all SBHC participants). Of these, three interviews were unusable, primarily due to low sound quality. The final interview sample (*N* = 50) was predominantly female (88%), with a mean age of 13.8 years old; 40% were of an Asian, African, or other minoritized ethnicity; and 56% had “very high” levels of psychological difficulties (Table 1). Compared with all SBHC participants, young people in the interview sample were significantly more likely to be female (*χ*^2^ = 9.7, *p* = 0.008), but were otherwise of a similar demographic profile. On average, interview participants attended 8.0 sessions of SBHC (*SD* = 2.4), which did not differ significantly from non-interview trial participants.

**Table 1:** Participant characteristics at baseline.

	Interview participants (*N* = 50)	All SBHC (*N* = 167)
**Gender**		
Female Male Other	44 (88%) 4 (8%) 2 (4%)	127 (76%) 37 (22%) 3 (2%)
Age (years)	13.8 (0.9)	13.7 (0.8)
Baseline Psychological Difficulties (SDQ-TD) Close to average Slightly raised High Very high	3 (6%) 11 (22%) 8 (16%) 28 (56%)	20 (12%) 33 (20%) 22 (13%) 87 (52%)
**School year**		
Year 8 Year 9 Year 10 Year 11	8 (16%) 22 (44%) 18 (36%) 2 (4%)	28 (17%) 79 (47%) 53 (32%) 7 (4%)
**Ethnicity**		
White Asian/Asian British African/Caribbean/Black British Mixed Other Missing	30 (60%) 7 (14%) 4 (8%) 9 (18%) 0 (0%) 0 (0%)	90 (54%) 16 (10%) 27 (16%) 29 (17%) 4 (2%) 1 (<1%)
**Disability**		
No disability Has a disability Missing	44 (88%) 5 (10%) 1 (2%)	142 (85%) 23 (14%) 2 (1%)

SBHC, School-based humanistic counselling.

#### Therapists

The SBHC intervention was delivered by a pool of 10 therapists (one therapist per school, excepting one school that had two therapists). Eight of the therapists were female, with a mean age of 44.8 years old (*SD* = 6.3, range = 25–63 years old). All of the therapists were of a white British ethnicity. All therapists were qualified to Diploma level (at least a two-year, part time training in counseling or psychotherapy), had been qualified for an average of 7.1 years (*SD* = 6.6, range = 1–25), and had received training in SBHC based on a treatment manual. Therapists were provided with regular supervision. Adherence to SBHC was independently rated.

### Standardized measures

The primary outcome measure used in the study was the Young Person’s CORE (YP-CORE), a self-report measure of psychological distress in young people (Twigg et al., 2016) and the most commonly used outcome measure in secondary school-based counseling in the United Kingdom (Cooper, 2013). Young people are asked to rate their psychological distress on 10 items using a five point scale (0–4), giving a total score between 0 and 40, with higher scores indicating greater levels of distress. The YP-CORE measure has been shown to be acceptable to young people, with a good level of internal consistency (Twigg et al., 2016; Blackshaw, 2021). Secondary standardized outcome meaures were the Strengths and Difficulties Questionnaire (Goodman, 2001), Revised Child Anxiety and Depression Scale (Ebesutani et al., 2012), Warwick-Edinburgh Mental Well-Being Scale (WEMWBS) (Tennant et al., 2007). In addition, we used the idiographic Goal-Based Outcome tool as a secondary measure, in which young people stated, and rated, their own personalized goals for therapy (Law and Jacob, 2015; Duncan et al., 2023).

Client satisfaction with treatment, as a secondary outcome measure, was evaluated using the 12-item Experience of Service Questionnaire (ESQ), a widely used measure with young people to assess satisfaction with treatment provision (Attride-Stirling, 2003). The ESQ asks respondents to, “Please think about the appointments you have had at this service or clinic,” and then to tick responses from a 2 (“Certainly true”) to 0 (“Not true”) scale, with the option of also ticking “?” (“Do not know”). Example items are “I feel that the people who saw me listened to me,” and “Overall, the help I have received here is good.” Testers were instructed to make it clear to the young people that, if they were in the SBHC condition, “service or clinic” referred to their therapy; and, if they were in the PCAU condition, it referred to “any pastoral care that they have had over the past 3 months, including contact with their pastoral care teacher.” Of the 12 items, nine have been found to form a “Satisfaction with Care” main factor (Brown et al., 2014). This factor has been found to be robust, and sensitive to differences between high and low scoring respondents. Scores on this dimension range from 0 to 18, with higher scores indicating greater satisfaction.

Therapy process was evaluated using the Barrett Lennard Relationship Inventory Form OS-40: T-S (Student Form) (version 3) (BLRI OS-40 T-S) and the Working Alliance Inventory Short Form (WAI-S) (Bhatti et al., 2024). The Barrett-Lennard Relationship Inventory (BLRI) is a family of measures based on Rogers (1957) theory of the necessary and sufficient conditions for therapeutic personality change: Empathic Understanding, Congruence, Level of Regard, and Unconditionality of Regard. Clients in the present study completed the OS-40: T-S (Student form) (v3) version (Barrett-Lennard, 2015). The WAI-S is a 12-item measure, adapted from the Working Alliance Inventory which assesses the collaborative and affective bond within the therapeutic relationship (Tracey and Kokotovic, 1989). It consists of three 4-item subscales: agreement on the goals of the therapeutic relationship (Goal subscale), collaboration on the tasks needed to achieve these goals (Task subscale), and the quality of the therapeutic relationship (Bond subscale). The WAI-S is the most used alliance measure with adolescents and has demonstrated good internal consistency within youth samples (Capaldi et al., 2016). In the present sample, Cronbach’s alpha = 0.94.

### Procedure

#### Recruitment

Recruitment to the trial was through the schools’ pastoral care teams. The teams were briefed on the study and, as a pre-screening stage, asked to identify potentially eligible young people. If young people expressed interest, their parents or carers were asked to provide written consent by a member of the pastoral care team. An assessor them met with the young person, formally assessed their eligibility, and (if eligible) invited them to provide written assent.

#### Randomization and masking

Trial participants were assigned (1:1) to one of two conditions: (a) school-based humanistic counseling along with access to usual pastoral care provision (SBHC group), or (b) access to usual pastoral care provision alone (PCAU group).

#### Intervention

SBHC is a manualized form of humanistic therapy [reference masked] based on evidence-based competencies for humanistic counseling with young people aged 11–18 years (British Association for Counselling and Psychotherapy, 2019). SBHC assumes that distressed young people have the capacity to address their difficulties if they can explore them with an empathic, supportive, and trustworthy counselor. SBHC therapists use a range of techniques, including active listening, empathic reflections, and inviting young people to express underlying emotions and needs. SBHC also included weekly use of the Outcome Rating Scale (Miller et al., 2003) so that the therapists could discuss with young people their progress during therapy. Sessions were delivered on an individual, face-to-face basis, and lasted 45–60 min. They were scheduled weekly over a period of up to 10 school weeks, with young people able to terminate counseling prior to this time point.

The counselors received, at minimum, 4 days of group training in SBHC, and were subsequently supervised by an experienced clinician throughout the trial. Adherence to SBHC was assessed by two independent auditors using a young person’s adapted version of the Person Centred and Experiential Psychotherapy Rating Scale (PCEPS-YP) (Freire et al., 2014; Ryan et al., 2023). All counselors exceeded the pre-defined adherence cut-point.

Participants in the SBHC group also had full access to their school’s *usual pastoral care support*, comprising the pre-existing services for supporting the emotional health and well-being of young people available within their school.

#### Outcome and process measurement schedule

The outcome measures (YP-CORE, SDQ, RCADS, WEMWBS, GBO Tools) were completed by all young people at baseline assessment and again at 6-weeks, 12-weeks, and 24-weeks post-baseline assessment by a tester who was blind to their allocation. At 12-weeks, participants were also asked to complete the ESQ. The BLRI OS-40 T-S and WAI-S were completed by all young people at 6-weeks.

#### Qualitative interviews

The aim of the interviews was to capture the informant’s sense of their agency in counseling, by offering them a format that they could use to describe helpful and hindering therapy *processes*—specific sequences of action leading to an outcome—along with generalized factors. The strategy for collecting this type of first-person qualitative accounts from clients around their experience of therapy was informed by guidelines for conducting end-of therapy client outcome interviews developed by Elliott (2002), Lilliengren and Werbart (2005), Cooper et al. (2015), and Sandell (2015). Key methodological elements adopted from these sources included a focus on helpful and hindering aspects of therapy, inviting clients to identify process sequences that contributed to outcomes, and preventing client overwhelm by integrating open-ended exploration of implicit and hard-to-articulate areas of experience into an overall structured framework. In addition to these features, a further innovative procedure involved visual mapping to support the identification of sequences, facilitate participant reflection on experience, and allow the interviewer to check and clarify their understanding of the information being provided by the interviewee. The interview schedule included a specific question about negative effects of counseling. To make it easier for informants to talk about hindering or harmful aspects of the counseling they had received, interviewers were not therapists, and were independent of the study.

Interviews were semi-structured and based around a topic guide (Supplementary material). The first, introduction section (approximately 5 min), invited the young person to say something about themselves, why they thought they were offered therapy, and whether they had spoken to people in their lives about their problems. The second, open-ended section of the interview (approximately 15 min), invited the young person to describe, in their own words, what they had found helpful or hindering in the therapy. To facilitate this, the young people were invited to fill out a blank “process map” (Supplemental Material: Process Map). This consisted of rows of four empty ovals, linked together with arrows, in which the young people could write: “What the counselor did,” “How you responded to this,” “Any changes as a result,” and “What happened next” (43 young people, 86%, completed at least one row of this map). The third, closed-ended section of the interview (approximately 15 min), asked the young people to confirm or disconfirm helpful and hindering factors that had been previously identified in the literature, as reviewed by the trial team, such as being helped to express feelings or gain new understanding, or being able to trust their counselor (Cooper et al., 2024).

The qualitative interviews were carried out on school premises, on average 5.5 weeks after the end of therapy (range: 1–16 weeks). There were four interviewers who carried out between two and 20 interviews each. The interviewers were experienced researchers from a national children’s charity. Transcription of the interviews was carried out by a professional transcription service, independent of the interviewers and data analysts.

#### Analysis of interviews

The procedure for rating the helpfulness of the interviews began with a codebook-style thematic analysis of the interview data [Braun and Clarke (2006, 2022)], conducted using NVivo v.11 and v.12 by a team led by Author 6 (see Cooper et al. (2024) for details of this procedure and findings). Author 6 then created “process narratives” for each of the 50 young people: summarizing what each young person concretely described as helpful (e.g., “Getting things off chest”) and unhelpful (e.g., “Silences awkward”) change processes in their therapy. Commonly-identified helpful and unhelpful processes of change were then written up, with descriptors, into a Process Analysis Codebook. Two independent Master’s level students then carried out a full, independent coding of all cases for helpful and unhelpful processes. A broad range of themes were identified through this procedure, that reflected both the client’s perception of helpful processes within sessions, and changes they had observed in their lives that they attributed to counseling. Outcome themes mentioned by clients included better communication, improved relationships, reduction in emotional distress, enhanced ability to participate in learning and school work, and improved coping strategies, resilience, self-control, confidence and self-acceptance.

Using a mixed methods interview analysis strategy developed by Di Malta et al. (2019), based on this coding of specific helpful and unhelpful change processes and outcome themes, each of the Master’s students was then asked to give, for each interview, an overall rating of “how helpful the counseling seems to have been for the young person.” The raters were instructed that, “This rating should be a number between 1 and 10: 1 = Not at all helpful, 10 = Extremely helpful.” Subsequent to the two raters’ scorings, the Author 6 also carried out a scoring of each interview, using the same scale. For our final helpfulness rating, we used the mean of ratings for each young person across the three raters. In the analysis, results, and discussion sections below, this numerical condenzation of qualitative accounts is described as the *helpfulness rating*. The final helpfulness ratings used the mean of ratings for each young person across the three raters. Raw correlations between raters ranged from 0.81 to 0.93. Inter-rater reliabilities were 0.94 (Cronbach’s Alpha) and 0.96 (McDonald’s Omega).

### Analysis

#### Preliminary analyses

As preliminary analyses, we first examined inter-rater reliabilities across the three ratings, using Cronbach’s alpha and McDonald’s omega. We then examined the distribution of helpfulness ratings across raters, and for the mean helpfulness scores; and examined the association of helpfulness scores with participant and intervention characteristics.

#### Multilevel regression analysis

In our primary analysis, we looked at the association between helpfulness ratings and YP-CORE scores. Multilevel analysis was appropriate for our data because young people were nested within counselors; and a multilevel approach takes into account the potential non-independence of nested data. In addition, for our outcome measures, we chose to focus on the slope of improvement over time (from 0 weeks, to 6 weeks, to 12 weeks, to 24 weeks) as this was considered the most veridical indicator of change associated with the intervention. Testing points, therefore, were nested within young people, giving us a three-level starting point for our outcome indicator models: counselor (*k*), young person (*j*), and testing point (*i*).

Procedures for the multilevel analyses followed guidelines proposed by and Singer and Willet (2003), Hox and Maas (2005), and Hox (2010), and were conducted using the software programme MLwiN (version 3.02) with the default iterative generalized least-squares (IGLS) method of estimation. Direct effects were entered into the model (Hox, 2010), even where they were not significant, so that the interactions could be meaningfully interpreted. To examine whether assumptions of normality and linearity had been met, graphs of level-1 and level-2 residuals by rank, and by fixed part predictions, were inspected—both after an initial model had been established, and for the final models (Hox, 2010). Variables were considered significant and retained if the coefficient was over 1.96 times the standard error. In addition, on introduction of each variable, we assessed goodness-of-fit, by a comparison of −2*loglikelihood ratios.

To develop our models, we first tested whether allowing our dependent variables to vary randomly by counselor, and by young person (for our outcome indicators), improved model fit. Next, for our outcome indicators, we introduced a *TIME* predictor into the model (weeks from baseline), which allowed for estimation of changes over time and the contribution of *TIME* to model fit. We then introduced our helpfulness rating into the model. This was the principal test of the association between YP-CORE and our helpfulness rating. However, we were primarily interested in the association between the helpfulness rating and changes in YP-CORE outcomes over time. Therefore, in these instances, we entered finally, and most importantly, the interaction between *TIME* and helpfulness rating. If helpfulness rating was associated with the YP-CORE score, we would expect to see significant coefficients here and a significant improvement in model fit.

#### Categorical analysis

To assess the relationship between our helpfulness ratings and YP-CORE scores, we also analyzed our data categorically. Here we used a median split on our helpfulness rating (median helpfulness rating = 7.33), to distinguish between those young people who were assessed as giving average or higher than average ratings of the helpfulness of the counseling, and those who were assessed at giving lower than average ratings. We then compared this, descriptively, against reliable improvement at 12 weeks on the YP-CORE (the principal outcome for the trial, and the one closest to the interview timepoint), using the indices established by Twigg et al. (2016): YP-CORE scores must change by more than 8.3 points (male, 11–13 years), 8.0 points (male, 14–16 years and female, 11–13 years) and 7.4 points (female, 14–16 years). This allowed us to see whether young people who showed reliable improvement tended to describe the intervention as helpful and vice versa, or whether there was a mismatch between evidence of reliable change and self-reported helpfulness.

#### Sensitivity analyses

We wanted to explore whether the relationship between our helpfulness rating and our symptom tracker would hold for all outcomes. Therefore, we also looked at correlations between our helpfulness rating and our other measures at 0–12 and 0–24 weeks: SDQ, RCADS, WEMWBS, GBO Tools, For satisfaction (ESQ), we used 12 week scores; and for our process variables (BLRI OS-40 T-S and WAI-S) we used 6-week ratings.

### Data availability statement

Publicly available datasets were analyzed in this study. Quantitative, participant level data for the ETHOS study (with data dictionary), and related documents (eg, parental consent form), are available from February 1, 2021, via the ReShare UK Data Service, https://reshare.ukdataservice.ac.uk/853764/. Access requires ReShare registration.

## Results

### Preliminary analyses

#### Ratings of helpfulness

Ratings of helpfulness from Raters A and B ranged from 1 to 10, and from Rater C (Author 6) from 1 to 9, with medians and modes of 8, 8, and 6, respectively. Mean scores were 6.7 (*SD* = 2.8), 6.5 (*SD* = 3.0), and 6.0 (*SD* = 2.0). Distribution for all three raters indicated a slight negative skew (skew statistic_Rater A_ *=* −0.71, SE = 0.33; skew statistic_Rater B_ *=* −0.58, SE = 0.34; skew statistic_Rater c_ *=* −0.65, SE = 0.34) but no evidence of significant kurtosis.

The mean helpfulness rating (subsequently referred to as “helpfulness rating”) per participant ranged from 1 to 9.7; with a median and modal score of 7.3; a mean of 6.4 (*SD* = 2.5); and, again, evidence of skew (skew statistic_Mean Rating_ *=* −0.64, SE = 0.34) but not kurtosis.

Helpfulness ratings did not correlate significantly with the young person’s age (*r* = 0.06, 95%CI = −0.22, 0.33). There was also no evidence of significant differences across gender [*F* (2, 49) = 1.2, *p* = 0.30], ethnicity [*F* (1, 49) = 0.05, *p* = 0.83], or disability [*F* (1, 48) = 3.86, *p* = 0.055]. However, the latter did show a trend for counseling to be rated as less helpful for young people identifying with a disability (*mean* = 4.4, *SD* = 3.0, *n* = 5) as compared with those without (*mean* = 6.7, *SD* = 2.4, *n* = 44). Given these generally non-significant associations, we did not include these demographic factors in subsequent analyses. There was a positive correlation between helpfulness ratings and the number of sessions that young people had (*r* = 0.36, *p* = 0.01).

The 10 counselors saw between four and seven clients. The mean helpfulness ratings for counselor ranged from 3.7 (*SD* = 3.1, *n* = 4) to 8.1 (*SD* = 0.7, *n* = 5). An ANOVA test did not find significant differences in mean helpfulness ratings across counselors [*F* (9, 49) = 1.5, *p* = 0.17]. However, 25.6% of the variance in helpfulness ratings could be accounted for at the counselor level.

#### Plotting helpfulness ratings against YP-CORE

**Figure 1:**
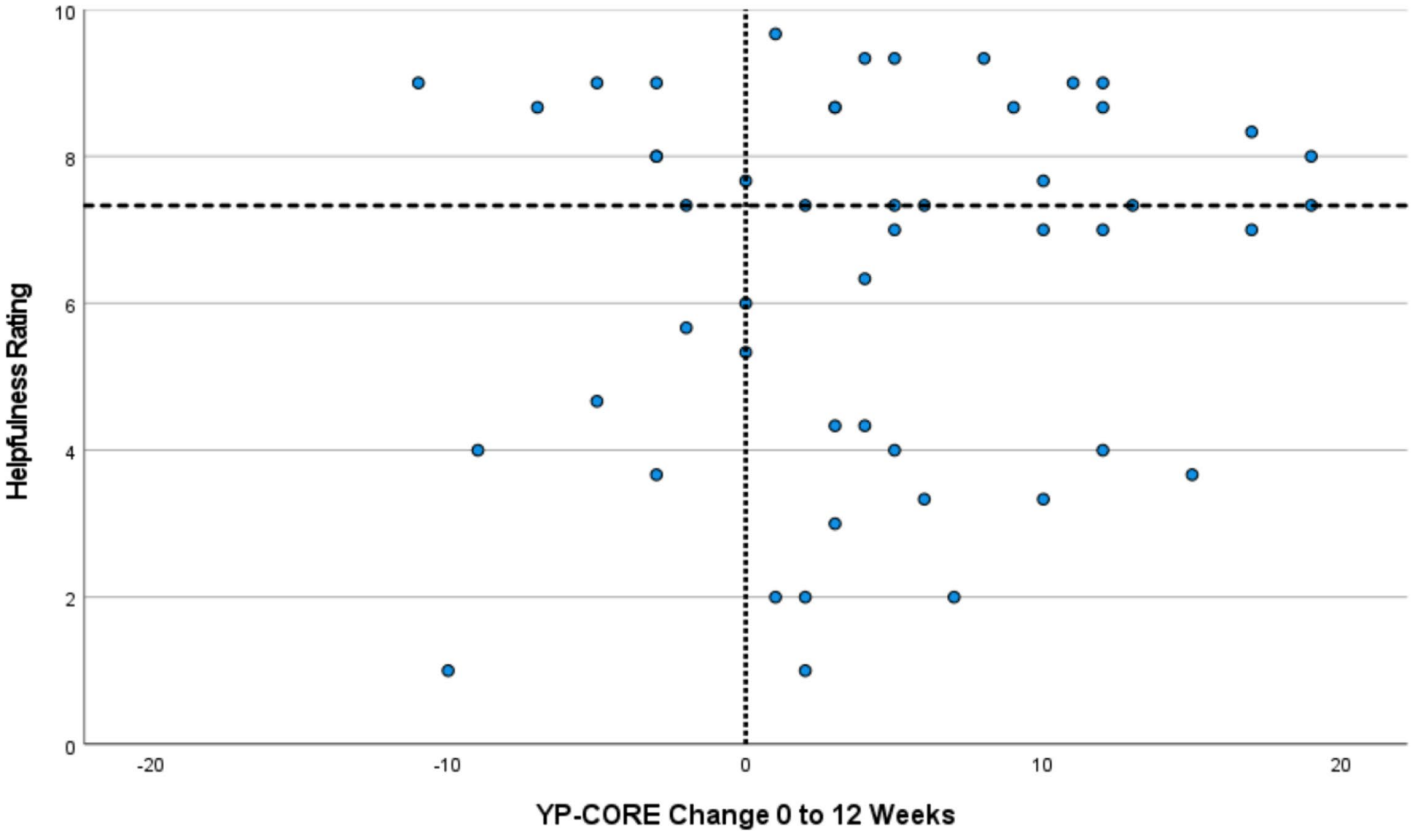
Helpfulness Ratings Against YP-CORE Change Scores from 0 to 12 Weeks. Reference line on X-axis indicates no change on YP-CORE. Reference line on Y-axis (*y* = 7.33) indicates median for helpfulness ratings. Plots in the top-right and bottom-left quadrants indicate a match between helpfulness ratings and YP-CORE change scores, while those in the top-left and bottom-right quadrants indicate a mis-match.

Figure 1 presents a scatterplot of helpfulness ratings against YP-CORE change from 0 to 12 weeks. The raw Pearson’s correlation was 0.14 (95% CI = −0.14, 0.40).

### Multilevel regression analysis

For the YP-CORE scores from 0 to 24 weeks, allowing the model to vary randomly by counselor reduced the −2*loglikelihood from 1366.5 to 1352.0, a − 2*ll ratio of *χ*^2^ = 14.5, *p* < 0.001. Random variation by young person further improved the −2*loglikelihood statistic to 1304.0, a − 2*ll ratio of *χ*^2^ = 48, *p* < 0.001. Variations in YP-CORE scores was 6.0% at the counselor level, and 45.6% at the young person level, with 48.4% variance at the individual outcome points. All three levels were therefore retained in the final model. As expected, baseline YP-CORE scores added further to model fit (fixed across counselors and young people): reducing the −2*ll statistic to 1256.0, a − 2*ll ratio of *χ*^2^ = 48, *p* < 0.001; and with a b-value of 0.75 (*SE* = 0.08). Adding the weeks indicator further improved model fit to 1249.5 (−2ll ratio of *χ*^2^ = 6.5, *p* = 0.01), with a *b*-value of −0.11 (*SE* = 0.04). This indicates that, for every week beyond the baseline assessment point, the YP-CORE score reduced, on average, by 0.11 of a point. Allowing this slope of improvement to randomly vary by young person (but not by counselor) led to further significant increases in model fit, down to 1227.5 (−2ll ratio of *χ*^2^ = 22, *p <* 0.001). Adding the helpfulness rating gave no additional benefit to model fit (−2ll statistic = 1226.5) and, crucially, adding the interaction between helpfulness rating and weeks did not add significantly to model fit (−2ll ratio of *χ*^2^ = −0.24); nor did the single parameter of −0.17 (SE = 0.17) for this interaction suggest that it significantly contributed to YP-CORE scores. To summarize, then, across the course of intervention and follow up, helpfulness ratings (i.e., client-defined outcomes) were not significantly associated with changes in YP-CORE scores.

### Categorical analysis

Table 2 shows frequencies of clients who demonstrated reliable change on the YP-CORE (12 weeks) against helpfulness ratings (median split). As this table indicates, 10 of the 16 young people who showed reliable improvement on the YP-CORE (63%) were at or above the median score of helpfulness, while 6 were below the median score (38%). Of the 34 young people who did not show reliable improvement on the YP-CORE, 18 were below the median score of helpfulness (53%), while 16 were at or above the median score (47%). In total, therefore, 28 of the young people (56%) had a helpfulness rating that corresponded with their improvement on the YP-CORE, while 22 (44%) showed paradoxical outcomes (discrepancy beween outcome status defined by a symptom measure, and one derived from the interview carried out with the client after completion of therapy). Figure 1 provides a visual display of the data considered in this analysis, with discrepant or paradoxical outcome profiles located in the top-left and bottom-right quadrants. It is possible to see that, in several cases, qualitative and quantitative outcomes sources have produced quite strikingly different outcome positioning, particularly in the lower right quadrant (succcesful outcome on the basis of YP-CORE data, alongside unsuccessful outcome based on interview data).

**Table 2:** Helpfulness rating against outcome and process scores.

	Correlation (*r*)	95%CI	*n*	*p*
YP-CORE 0–12	0.14	−0.14, 0.40	50	0.33
YP-CORE 0–24	−0.08	−0.33, 0.24	49	0.59
SDQ 0–12	0.25	−0.03, 0.49	50	0.08
SDQ 0–24	−0.05	−0.33, 0.24	48	0.74
RCADS 0–12	0.22	−0.06, 0.47	50	0.12
RCADS 0–24	0.18	−0.10, 0.44	49	0.21
WEMWBS 0–12	0.30	0.02, 0.53	50	0.04*
WEMWBS 0–24	0.05	−0.27, 0.35	41	0.77
Goals 0–12	0.29	0.01, 0.52	50	0.04*
Goals 0–24	0.31	0.03, 0.55	49	0.03*
Satisfaction – ESQ	0.45	0.18, 0.67	42	0.003**
Empathy – BLRI	0.42	0.15, 0.62	49	0.003**
Congruence – BLRI	0.35	0.08, 0.58	49	0.01*
Unconditionality - BLRI	0.09	−0.20, 0.36	49	0.56
Regard – BLRI	0.28	0.00, 0.52	49	0.05*
Task – WAI	0.40	0.12, 0.62	46	0.006**
Bond – WAI	0.39	0.11, 0.61	46	0.007**
Goals – WAI	0.39	0.11, 0.61	46	0.007**

*p < 0.05, **p < 0.01.

0-12 = change from baseline to 12 week testing point, 0–24 = change from baseline to 24 week testing point. Positive scores indicate reductions in distress (YP-CORE, SDQ, RCADS); or improvements in wellbeing (WEMWBS), satisfaction (ESQ), or therapeutic relationship (BLRI, WAI).

YP-CORE, young person’s clinical outcomes in routine evaluation measure; SDQ, strengths and difficulties questionnaire – total difficulties; RCADS, revised child anxiety and depression scale – total score; WEMWBS, Warwick and Edinburgh Mental wellbeing scale; Goals, goal based outcome tool – mean goal score; Satisfaction ESQ, experience of service questionnaire (12 week only); BLRI = Barrett-Lennard relationship inventory, WAI, working alliance inventory (short form).

### Sensitivity analyses

Raw correlations for mean helpfulness rating against young people’s outcome and process scores are presented in Table 3. Mean helpfulness ratings did not correlate significantly with change from baseline to 12 weeks, or baseline to 24 weeks, on any of the measures of psychological distress: YP-CORE (*r*_0-12 weeks_ = 0.14, *r*_0-24 weeks_ = −0.08), SDQ-TD (*r*_0-12 weeks_ = 0.25, *r*_0-24 weeks_ = −0.05), and RCADS (*r*_0-12 weeks_ = 0.22, *r*_0-24 weeks_ = 0.18). There was a significant, moderate correlation between mean helpfulness ratings and improvements in wellbeing (WEMWBS) for 0–12 weeks (*r* = 0.30) but not 0–24 weeks (*r* = 0.05). Improvements in goal attainment (GBO tool) correlated significantly and moderately with mean helpfulness ratings for both 0–12 weeks (*r* = 0.29) and 0–24 weeks (*r* = 0.31). There was a large correlation between satisfaction with counseling (ESQ) and mean helpfulness ratings at 12 weeks (*r* = 0.45). BLRI ratings of empathy, congruence, and regard (6-week midpoint) all showed significant, moderate to large correlations with mean ratings of helpfulness (*r*s = 0.42, 0.35, 0.28). There were also significant, moderate to large associations between the alliance subscales and mean helpfulness ratings (*r*_goal_ = 0.39, *r*_task_ = 0.40, *r*_bond_ = 0.39).

**Table 3:** YP-CORE by helpfulness ratings categorical outcomes.

YP-CORE reliable improvement (*n*, row %)	Helpfulness rating		
	Above or at median	Below Median	Total
Yes	10 (63%)	6 (38%)	16
No	16 (47%)	18 (53%)	34

Median helpfulness score = 7.33.

## Discussion

The aim of the study was to examine the extent to which perceptions of therapeutic helpfulness, from client interview data, would relate to other outcome measures in humanistic counseling for young people. We analyzed the relationship between outcome, as assessed by a standardized self-report measure, YP-CORE, and young people’s experiences of helpfulness in counseling, as assessed by ratings of post-counseling interviews. Our results showed that of the 50 participants, as many as 44% of the young people had a helpfulness rating that did not correspond to improvement as assessed by the outcome measure YP-CORE. More specifically, 38% of those who showed reliable improvement on the YP-CORE were below the medium score of helpfulness, whereas 47% of those who did not show reliable improvement on the YP-CORE were at or above the median score of helpfulness. Based on these results, treatment failure or success is not a straightforward matter. These results are consistent with findings of other studies that have similarly reported differences between outcomes recorded through self-report measures and those based on qualitative interviews (McElvaney and Timulak, 2013; Bloch-Elkouby et al., 2019; De Smet et al., 2019, 2020a,b, 2021a,b, 2024; Desmet et al., 2021a). Taken together, the evidence from these studies as a whole suggest that the phenomenon that we have characterized as paradoxical outcome is a robust pattern that has been identified in different samples using different data collection and analysis strategies. The present study adds to this body of knowledge by offering a method through the which the prevalence of paradoxical outcome can be estimated.

There are several possible explanations to the discordance between self-report measures and interview data in the present results. The client interviews involved three phases particularly developed to help young people talk, which may have facilitated access to aspects of their treatment processes, and implicit outcome criteria, not addressed within the primary outcome measure. The possibility that lack of correspondence between outcomes as assessed by client interviews, and those generated by analysis of pre- and post-counseling YP-CORE, could be due to lack of validity or coherence in the qualitative material is not supported: helpfulness ratings derived from qualitative data showed a consistent moderate to large correspondence with process measures (Barrett-Lennard Relationship Inventory BLRI, the Working Alliance Inventory WAI-S), improvements in goal attainment (GBO Tool), and satisfaction with counseling (ESQ), suggesting that these sources may capture similar aspects of processes and change occurring for clients.

Our findings both support and extend previous research that has shown meaningful differences between outcome criteria reflected research measures, and the ways that clients and other stakeholders evaluate the effectiveness of therapy (Chevance et al., 2020; Bear et al., 2021; Krause et al., 2021; Axelsdóttir et al., 2022; Morton et al., 2022; Amin Choudhury et al., 2023). In the present study, the primary outcome measure—YP-CORE—was designed to capture degree of distress/well-being being experienced by a respondent, in relation to their life as a whole. By contrast, the methods for assessing outcome and process in the present study that yielded broadly convergent indications of outcome - interviews, ESQ, GBO Tool, WAI-S and BLRI - were all explicitly anchored in the client’s experience of counseling. It is possible that YP-CORE was more sensitive to the impact of extra-therapy sources of stress or support, whereas the other instruments were more sensitive to change arising specifically from counseling. This distinction does not appear to have been highlighted in the existing counseling and psychotherapy literature. It represents a factor that may be particularly salient in relation to the organizational context of the present study, in which counseling was provided in a situation in which there already existed other accessible sources of support, for instance from teachers or educational psychologists.

Evidence of a large correlation between satisfaction with counseling (ESQ) and mean helpfulness ratings (*r* = 0.45), both collected post-therapy, is consistent with a response shift perspective that predicts that the client’s understanding of their presenting problem and how it has changed becomes more differentiated and accurate over the course of therapy (Golembiewski et al., 1976; Howard and Daily, 1979; Bulteau et al., 2019). The concordance between these retrospective judgments supports the conceptualization of Flückiger et al. (2019) concerning the reliability of outcome assessment based on subjective perception of change. The correlation between helpfulness ratings and goal attainment (0–12 weeks, *r* = 0.29; 0–24 weeks, *r* = 0.31) may reflect the fact that both assessment approaches are grounded in the client’s personal criteria for change. Sigificant levels of correlation between helpfulness ratings and scores on both the BLRI and WAI-S may be attributed to the content (i.e., client accounts of what was helpful or hindering in counseling) underlying the helpfulness ratings. A substantial cluster of hindering process narratives generated by clients referred to alliance ruptures or more general failure to develop mutual understanding with the counselor (Ralph and Cooper, 2022; Pattison and Cooper, 2024).

The scatterplot (Figure 1), which visually displays the distribution of cases across a two-dimensional space defined by YP-CORE scores and helpfulness ratings derived from qualitative interviews, is suggestive of a range of interpretations that may have heuristic value in relation to the design of further studies. The spread of cases across the two-dimensional space makes it possible to identify some cases in which the degree of paradoxical outcome was minimal, and others where the disparity was extreme. It could be valuable in further research to explore in more detail what is happening in these extreme cases. The quadrant lines in Figure 1 reflect a decision to divide the sample along the lines of positive vs. negative YP-CORE change, and median split of helpfulness ratings. Other strategies for dividing the sample could have been deployed, for example using clinical and reliable change indices for YP-CORE data, and developing a similar cut-off that differentiated between good and poor qualitative outcomes. However, it is not possible to imagine, given the distribution of cases, any quadrant lines that would eliminate paradoxical cases.

We believe that this study makes a unique contribution to research and practice in relation to understanding failure and success in therapy, by documenting the limitations of placing too much reliance on outcome data from symptom measures administered pre- and post-therapy alone. This investigation has a high level of ecological validity through being grounded in a large-scale study of the effectiveness of counseling in a real-world setting, and through the availability of published analyses of other aspects of the main study, that enable a deeper understanding of contextual factors. Further strengths of the study are the development of an interview strategy that builds on previous work around qualitative outcome assessment, and the use of a mixed methods technique for numerical representation of qualitative themes.

Limitations of the study are associated with its status as a secondary analysis of data from a primary study that was not designed with the intention of analyzing paradoxical outcomes. To enable a more meaningful exploration of paradoxical outcome, it would have been useful to have included interviews with control group (treatment as usual) participants, and to have been able to ask participants, at the end of their interview, for their own numerical summary rating of how much they had benefitted from counseling and then compare their ratings with those generated by data coders. It would have been valuable to have collected additional information around the factors that influenced clients to participate in interviews. Futher insights would certainly have emerged if we had been able to conduct additional interviews with clear-cut paradoxical outcome cases to learn about how participants made sense of apparently discrepant outcome profiles, and carry out intensive case analysis of such cases. In the light of these limitations we have intentionally tried to avoid reading too much into our findings.

Our hope is that this study will lead to other work on the incidence and structure of paradoxical outcomes in large data sets, with the aims of both generating new insights and building a more complete understanding of the meaning and implications of the qualitative and case-based research literature that already exists around this topic. It could be particularly fruitful to examine the nature and extent of paradoxical outcome in clients who identify themselves as belonging to minority and racialized communities. It seems possible that widely-used standardized symptom measures may not offer a good fit with everyday ideas about therapy success and failure that exist within such cultural groups. Clients from marginalized communities may also have good reason for lacking trust in the motives of researchers, or in the ways that their personal information could be used. Re-aligning therapy outcome procedures to be more responsive to such beliefs, could form a key step in moving toward decolonized and social justice-oriented forms of therapy practice.

## Conclusion

The findings of this study suggest that, when evaluating whether treatment has been successful or unsuccessful, it is problematic to place too much reliance on evidence from self-report symptom measures. This position is increasingly acknowledged within the psychotherapy and mental health community, through initiatives that exhort the profession as a whole to reconceptualize and reconsider how assessment of outcome is understood and carried out (Sandell and Wilczek, 2016; Rønnestad et al., 2019; Devji et al., 2020; Chui et al., 2021; Fried et al., 2022; Krause et al., 2022), including proposals for incorporating various types of qualitative outcome tools into routine outcome and feedback systems and outcome studies (Hill et al., 2013; McLeod, 2017; Roubal et al., 2018; Ogles et al., 2022). In order to take this agenda forward, we suggest that it will be important to gain a better understanding of what different evaluation strategies have to offer, and the strengths and limitations of different ways of combining evidence from different sources. It is also necessary to develop ways of including meaningful client participation and collaboration in decisions on whether therapy has been, or is on track to be, successful or unsuccessful (Aschieri et al., 2016; Catchpole, 2020).

We would like to offer some additional reflection that goes beyond the findings of the present analysis, and considers the wider social implications of the phenomenon of paradoxical outcome. The United Kingdom at the present time is faced by a mental health crisis in young people. Counseling in schools represents a potentially valuable strategy for addressing such problems at an early stage. The qualitative evidence generated by the ETHOS trial, in the form of interviews with young people and their parents/carers, arrived at two main conclusions: (a) counseling was widely appreciated and perceived as helpful, and (b) interviewees identified readily achievable ways of making it more helpful (Longhurst et al., 2022; Cooper et al., 2024). By contrast, the quantitative evidence suggested that counseling was only marginally more effective than the emotional support systems that already existed in the schools that took part in the study, and came at additional cost (Cooper et al., 2021). Within both the psychotherapy research community, and policy-making contexts, the former source of evidence is largely disregarded, while the latter is privileged.

Discrepancies between estimates of the success of therapy, derived from qualitative interviews and analysis of change in symptom scores, can be treated as a technical challenge that can be resolved through designing better measurement procedures. By contrast, when such discrepancies as regarded as *paradoxical,* resolution is only possible through consideration of underlying assumptions. Two key aspects of the conceptualization of psychotherapy outcome may need to be re-examined. First, the assumption that outcome can be adequately understood in terms of a single dimension, ranging from successful to unsuccessful, may not be appropriate. Second, there may be some analytic traction in viewing the existence of paradoxical outcome in psychotherapy research as an example of “epistemic privilege” (Greenhalgh et al., 2015; Byrne, 2020). An evidence hierarchy that assumes that one source of knowledge (quantitative data from large samples) is more valid than another (accounts of lived experience) may be operating as a barrier to understanding.

The issues raised by the existence of discrepant or paradoxical psychotherapy outcomes are similar to those identified by McAdams (1995) in his critical review of several decades of research on personality and individual differences. McAdams (1995) arrived at a position of viewing self-report measures as representing “the psychology of the stranger”: an understanding of how a person might be understood, in terms of broad patterns of behavior, by someone who does not really know them. By contrast, the kind of relational connection and collaboration that psychotherapy strives to achieve affords access to the narratives of a person’s life: “a more detailed and nuanced description of a flesh-and-blood, in-the-world person, striving to do things over time, situated in place and role, expressing herself or himself in and through strategies, tactics, plans, and goals” (McAdams, 1995, p. 366). In a professional landscape increasingly dominated by ultra-brief and AI-assisted psychotherapy, an approach to evaluating success and failure in psychotherapy solely or predominantly through a stranger’s gaze does not seem to us to be morally or ethically the right choice, no matter how convenient it may seem from an administrative or scientific perspective. As in other areas of life, the emergence of a paradox acts as a stimulus to fresh thinking. We believe that resolution of the outcomes paradox requires psychotherapy researchers to attend not only to important technical and methodological issues that surround this topic, but also to how these issues align with a commitment to social justice.

## Data availability statement

Publicly available datasets were analyzed in this study. This data can be found here: Quantitative, participantlevel data for the ETHOS study (with data dictionary), and related documents (eg, parental consent form), are available from February 1, 2021, via the ReShare UK Data Service, https://reshare.ukdataservice.ac.uk/853764/). Access requires ReShare registration.

## Ethics statement

The studies involving humans were approved by the University Ethics Committee of the University of Roehampton (reference PSYC 16/227), on August 31, 2016. The studies were conducted in accordance with the local legislation and institutional requirements. Written informed consent for participation in this study was provided by the participants’ legal guardians/next of kin.

## Author contributions

JM: Writing – review & editing, Writing – original draft, Conceptualization. ES: Writing – review & editing, Writing – original draft, Conceptualization. HO: Writing – review & editing, Writing – original draft, Conceptualization. SS: Methodology, Data curation, Writing – review & editing, Writing – original draft. PP: Methodology, Data curation, Writing – review & editing, Writing – original draft. MC: Data curation, Writing – review & editing, Writing – original draft, Project administration, Methodology, Investigation, Funding acquisition, Formal analysis, Conceptualization.
