# Supplementary material for: How do we know whether treatment has failed? Paradoxical outcomes in counseling with young people
Source: Front Psychol. 2024 Jun 4;15:1390579. doi: 10.3389/fpsyg.2024.1390579 (PMC11184953; doi:10.3389/fpsyg.2024.1390579)

# Paradoxical Outcomes in Young People

# Supplemental Material

**Supplementary Material 1: Patient and Public Involvement**

A panel of young people (drawn from the Young Person’s Advisory Group [YPAG] at the National Children’s Bureau, NCB) and a panel of parents and carers (drawn from the Parent and Carers Advisory Group at NCB) met face-to-face with the researchers at the start of the project—with follow-up email consultation—to advise on the development of methods. Involvement of these panels was at the level of ‘interactive advice’ and ‘light consultation’^1^, with guidance on the choice of outcome measures, the development of participant-facing materials, and strategies for reducing the burden of the research on participants. Self-nominating representatives from both panels then joined the Trial Steering Committee (TSC). This met, face-to-face, throughout the duration of the study; advising on all elements of study design, progress, and dissemination. The young people’s and parent/carers’ involvement in the TSC was supported by an NCB facilitator, who met with them prior to the start of TSC meetings—and accompanied them during the meetings—to ensure that they understood the TSC’s aims and the issues emerging, and could express their views. Members of the YPAG were aged 13-18 years old, interested in issues of mental health and wellbeing, and not involved as participants in the trial. They were reimbursed for their time.

^1^Sellars, E., Pavarini, G., Michelson, D., Creswell, C., & Fazel, M. (2020). Young people’s advisory groups in health research: scoping review and mapping of practices. *Archives of Disease in Childhood*, archdischild-2020-320452. https://doi.org/10.1136/archdischild-2020-320452

# Supplemental Material 2. Interview Topic Guide

**Young People’s Interview Topic Guide**

**Note for researcher: use this topic guide flexibly, it is not a script.**

**Questions are written as a guide and researcher should use and probe as appropriate.**

**Our overall aim is to develop a *rich* and *in-depth* understanding of the participants’ experiences, perceptions and any process of change.**

**Use probes and follow-up questions to help participants ‘unpack’ these as far as they are able and willing to.**

- Introduce self, thank participant for time and speaking to us,
- Go through information sheet and consent form again – reminder of purpose of research, confidentiality and reporting:
  - This research is looking to evaluate the effectiveness of counselling in reducing distress in young people. The key questions we want to answer are:
    - What did you find helpful and unhelpful in School Based Humanistic Counselling?
    - What did you see as any process(es) of change in school based humanistic counselling?
  - This interview is not about issues that you received counselling for
  - There are no right or wrong answers
  - If you want to stop at any point or don’t want to answer a question, then just say so. The important thing is that you feel comfortable
  - What you say to me during this interview will not be shared with teachers, other students, your parents or your counsellor unless you say something that makes me think that you or someone else is at risk of serious harm. I will have to share this with the lead for the project at University of XXX.
  - Some of what you say may be used in reports and publications, but nobody will know it is you who said it
- Check permission to record before starting digirecorder:
  - The recording will only be accessed by the research team for the purpose of the research. It will be transcribed confidentially after the interview to allow analysis. The recording and transcript will be stored securely
- Ask if have any questions about what said or the interview
- Check happy to take part and for the interview to start

*Section One: Background Information [keep to five minutes]*

1. Please start by saying a bit about yourself, such as how old you are, where you live, who you live with
   1. How long have you been at school?
   2. What is your favourite subject?
   3. Who’s your favourite teacher?
   4. Tell me about your friends, do they go to this school?

**If feel young person is comfortable, move on to next questions. Remind them that interview is about counselling and any process of change from it.**

1. What did you think when you were offered counselling? (Prompt: did you think it would help you?)
   1. Did you know what counselling was?
   2. What did you expect from the counselling?
   3. What did you want from the counselling?
2. Have you spoken to anyone outside of your family in the past about things that have caused you distress or which you were worried about?

*[If struggling, use following prompts]*

- 1. Who did you speak to?
  2. How did you do this – e.g. did you phone a helpline or chat online to someone, go to see your GP, go to a service for children and young people?
  3. Are you still using any of these services?

1. *Section Two: Your experience of counselling [fifteen minutes]*

I’d like us to draw a model of your experience of counselling please. We have a prepared model here, but you can use a blank piece of paper if you prefer. I can write it for you if you don’t want to [*Allow up to 10 mins for completion]*

1. When was your last session of counselling?
2. Thinking back to all the sessions you had in school with the counsellor, can you talk me through the following and add to the model:
3. Was there anything the counsellor did that you found helpful or unhelpful? [e.g. counsellor seemed friendly; counsellor listened; they seemed to care or take young person’s worries seriously] *Please fill in the* *first column – what counsellor did*
4. How did you respond to this? [e.g. any feelings or actions you took] *Please fill in the second column – how you responded*
5. Any changes, good or bad?[Prompt: this could be changes in your own feelings about yourself, others or your situation; your behaviour or your actions and/or your relationships with others] *Please fill in the third column*
   1. Were these changes helpful or unhelpful? WHY?
6. [For example, why did it make you feel/do this and why was this helpful/unhelpful to you]
7. In the fourth column, please tell me if anything happened as a result of any of these changes [e.g. felt able to join in more at school, able to concentrate/grades improved]
8. *Fill in* *fourth column – what happened next*
9. Looking at your model, I’d like to talk a bit more about whether you see any links between the counselling you received and any changes you’ve identified:
   1. For each change you have mentioned, was there anything specific about the counselling that caused this?
   2. Could anything else have caused these changes? [Prompt: What else was going on in your life at the time, e.g. getting help from somewhere else?]
10. *Section Three: In-depth exploration of process of change [fifteen minutes]*

The following questions should be used flexibly, depending on what has been said in relation to the model.

If feel participant has not mentioned activities listed, then probe.

There has been research on the process of change from school-based counselling before and what people have found helpful or unhelpful. I want to explore your experiences in relation to what this research found.

1. I wanted to ask about some specific things that your counsellor might have done with you, and whether or not you found them helpful or unhelpful. If they were helpful or unhelpful, it would be great if you could say why. So **did or didn’t they**:
   1. **listen carefully** to you? (prompt [if yes or no], Was that helpful/unhelpful? Why?)
   2. **understand** you (prompt [if yes or no], Was that helpful/unhelpful? Why?)
   3. **help** you **express your feelings** (prompt [if yes or no], Was that helpful/unhelpful? Why?)
2. Now I want to ask you some specific things about the kind of person your counsellor might have been with you, whether or not you experienced this, and whether or not it was helpful.
3. Again, if it was helpful or unhelpful, it would be great if you could say why – what kind of effect it had.
4. **Did you or didn’t you**:
   1. feel you could **trust** them (prompt [if yes or no], What was the effect of that?)
   2. feel they were **friendly**
   3. **consistent** (always there, in same place at same time each week, acting in same way each week)
   4. **could you depend** on them
   5. **accepting** of what you had to say
   6. **cared** about you or your views
   7. **independent** (e.g. someone who was separate from the school and your family)
   8. **confidential**
5. Now I’d like to ask you a bit about what YOU did in counselling, and whether or not that was helpful or unhelpful. Remember there are no right or wrong answers. For example, did you or didn’t you talk about how you really felt/what you were experiencing
   1. Was this helpful or not?
6. [Prompts a-e of this question are only to be used if mentioned by the participant at question 9 – probe on relevant helpful things, but if only unhelpful mentioned then go to prompt f] I’d like to ask you about **why** these **things were helpful [e.g. talk/be listened to]** – **how and why did it help you**.
7. [Prompts for if struggling] For example, if you did talk about your genuine feelings, did this help you:
   1. Get things off your chest and feel like you were unburdening yourself of things (prompt: if so, **Why was that useful/how did it help**)
   2. Develop your communication and relationship skills, and feel more able to talk to others outside of counselling about what you’re really feeling (prompt: if so, Why was that useful/how did it help)
   3. Feel more accepting about yourself and what you have been experiencing (prompt: if so, Why was that useful/how did it help)
   4. Understand more about yourself, others, and your situation and why you do things (prompt: if so, Why was that useful/how did it help)
   5. Find ways of doing things that work out better for you (prompt: if so, Why was that useful/how did it help
   6. **If you DIDN’T find it helpful to** [talk about how you were feeling], why do you think this was? How did this make you feel? Could anything have been done differently?
8. Did the counselling give you guidance and advice? Was this in any way helpful or unhelpful?
   1. If you received guidance/advice, how did this make you feel?
   2. Can you talk about whether this had any effect on your ways of coping and stress?
   3. How do you know this was the counselling and not something else?
   4. **If it didn’t** give you advice or guidance, how did this make you feel?
9. Do you think that the counselling had any effect or not on your emotional distress? Can you say why or why not?
   1. If it did change your levels of emotional distress did this change, in any way, your engagement in school? How?
10. Do you think the counselling led to any adverse effects? This means it could have been harmful/negative to you.
    1. [If yes], in what way were you affected by it?
    2. [If yes], how did the counselling do that?
11. [If not discussed] Is there anything you would improve about the counselling?
12. Is there anything else that you want to say about your experience of counselling, the counsellor, or how you feel following the sessions?

Close interview and thank participant.

# Supplemental Material 3. Process Map


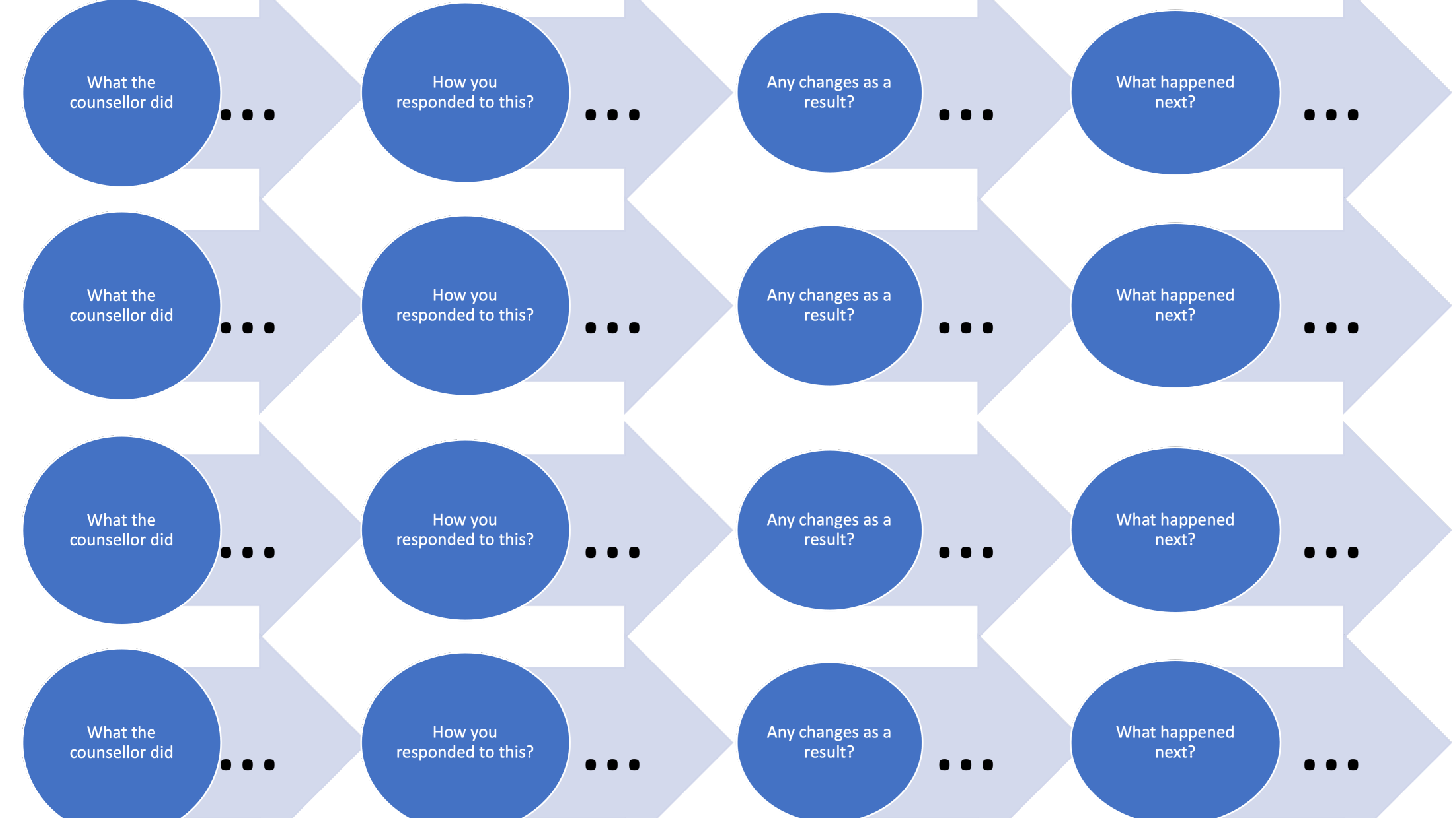

Supplement: Supplementary file 1 [file Table_1.DOCX]
